# Is there a single best estimator? Selection of home range estimators using area-under-the-curve

**DOI:** 10.1186/s40462-015-0039-4

**Published:** 2015-04-16

**Authors:** W David Walter, Dave P Onorato, Justin W Fischer

**Affiliations:** U.S. Geological Survey, Pennsylvania Cooperative Fish and Wildlife Research Unit, 403 Forest Resources Building, University Park, PA 16802 USA; Fish and Wildlife Research Institute, Florida Fish and Wildlife Conservation Commission, 298 Sabal Palm Road, Naples, FL 34114 USA; United States Department of Agriculture, Animal and Plant Health Inspection Service, Wildlife Services, National Wildlife Research Center, 4101 LaPorte Avenue, Fort Collins, CO 80521 USA

**Keywords:** Area-under-the-curve, Brownian bridge movement models, Global positioning systems, Home range estimators, Isopleth, Kernel density, Schedule

## Abstract

**Background:**

Global positioning system (GPS) technology for monitoring home range and movements of wildlife has resulted in prohibitively large sample sizes of locations for traditional estimators of home range. We used area-under-the-curve to explore the fit of 8 estimators of home range to data collected with both GPS and concurrent very high frequency (VHF) technology on a terrestrial mammal, the Florida panther *Puma concolor coryi*, to evaluate recently developed and traditional estimators.

**Results:**

Area-under-the-curve was the highest for Florida panthers equipped with Global Positioning System (GPS) technology compared to VHF technology. For our study animal, estimators of home range that incorporated a temporal component to estimation performed better than traditional first- and second-generation estimators.

**Conclusions:**

Comparisons of fit of home range contours with locations collected would suggest that use of VHF technology is not as accurate as GPS technology to estimate size of home range for large mammals. Estimators of home range collected with GPS technology performed better than those estimated with VHF technology regardless of estimator used. Furthermore, estimators that incorporate a temporal component (third-generation estimators) appeared to be the most reliable regardless of whether kernel-based or Brownian bridge-based algorithms were used and in comparison to first- and second-generation estimators. We defined third-generation estimators of home range as any estimator that incorporates time, space, animal-specific parameters, and habitat. Such estimators would include movement-based kernel density, Brownian bridge movement models, and dynamic Brownian bridge movement models among others that have yet to be evaluated.

**Electronic supplementary material:**

The online version of this article (doi:10.1186/s40462-015-0039-4) contains supplementary material, which is available to authorized users.

## Background

Recent advances in global positioning system (GPS) technology for monitoring wildlife have revolutionized data collection for spatial analysis of movements, home range, and resource selection. These datasets acquired with GPS technology are more copious and locations are more precise when compared to locational data collected using very high frequency (VHF) systems. Although published studies have reported on the reliability of home range estimators using datasets collected with VHF technology [1,2], few have identified the potential issues of estimating home ranges using the expansive datasets often collected with GPS technology [3,4]. Considering most traditional estimators of home range were developed for VHF datasets that typically consist of fewer than 100 locations and presumed to not be correlated in space and time, researchers are challenged with deciphering the most appropriate methods to estimate size of home range using GPS data sets that are often auto-correlated with extremely large sample sizes for a defined sampling period.

Concurrent with advances in GPS technology, alternative methods for estimation of home range have been developed to accommodate large numbers of auto-correlated relocations from GPS datasets. Amongst these are first-generation methods such as kernel density estimators that have proven capable of providing home ranges using large GPS datasets (KDE; [3-5]), although selection of the appropriate bandwidth for KDE is not always straightforward. Subsequent improvements in bandwidth selection have been developed for KDE using second-generation methods (e.g. solve-the-equation, plug-in; [5-7]). Local convex hull nonparametric kernel method (LOCO), which generalizes the minimum convex polygon method, was investigated for identifying hard boundaries such as water bodies and roads in home ranges but has not been evaluated with GPS datasets with >1,000 locations [8-10]. Brownian bridge movement models (BBMM) and dynamic Brownian bridge movement models (dBBMM) are ideal for GPS datasets when locations are collected in rapid succession – short time intervals between fix attempts producing locations that are serially correlated – because these methods incorporate time between successive locations into the utilization distribution estimation (hereafter referred to as third-generation estimators; [11-13]). An additional third-generation estimator, biased-random bridge, has been suggested as a movement-based KDE through location interpolation that include habitat-specific movement vectors [14,15]. Although these methods have all assisted with deriving more accurate estimation of home range with GPS datasets, developing a framework to assist with selecting the most appropriate estimator for each unique dataset is lacking in the literature.

Traditionally, the suggested estimator of home range was based on simulated datasets [16,17] that researchers cited as the sole justification for selecting KDE to estimate home range. Researchers would not attempt to identify the most appropriate estimator for their dataset but arbitrarily choose one to apply across all datasets. Coupled with the increased popularity of the freely available, open-source software Program R (R Foundation for Statistical Computing, Vienna, Austria; hereafter referred to as R), a method to determine the selection of an appropriate estimator for estimation of home range for wildlife is warranted.

Recently, area-under-the-curve (AUC) was assessed as an analytical means of choosing the most appropriate estimator of home range for an avian and mammalian species [10]. The AUC provides a single relative metric of goodness-of-fit by assessing how location-specific data fit the contours or isopleths of the estimator. Although the “best” estimator has been attempted using simulated datasets, AUC is a more intuitive metric of fit and is able to provide a relative metric of best estimator based on location collection schedules, distribution of points over the landscape, and inherent species-specific differences in movements [18]. To assess the use of AUC to help select appropriate estimators of home range, we used relocations collected on Florida panther (panther; *Puma concolor coryi*) with GPS technology and concurrent VHF technology to explore relationships between 8 estimators of home range. Specifically, our objectives were to: (1) determine if AUC differed for estimators of home range between locations collected with GPS versus concurrent VHF technology and (2) assess factors that influence AUC for estimators of home range across a range of individual animals using GPS technology.

## Methods

### Study area

**Figure 1:**
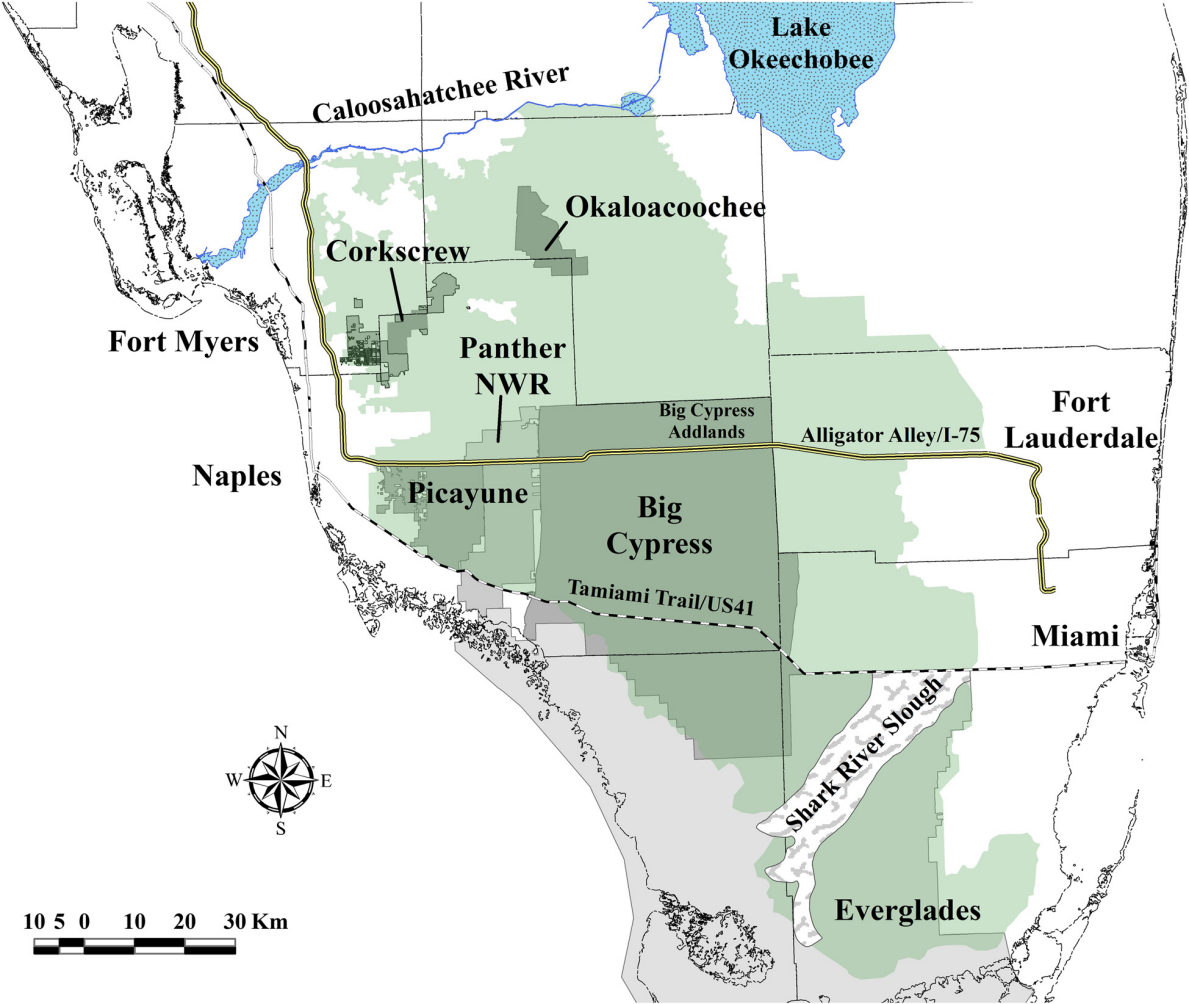
Map depicting the major public land holdings used in modeling of components that influence area-under-the-curve for estimators of home range for Florida panther in South Florida, USA. Key study area terms from south to north: Everglades, Everglades National Park; Big Cypress, Big Cypress National Preserve; Big Cypress Addlands, Additional Land units of Big Cypress National Preserve; Picayune, Picayune Strand State Forest/Fakahatchee Strand Preserve State Park; Panther NWR, Florida Panther National Wildlife Refuge; Corkscrew, Corkscrew Regional Ecosystem Watershed; and Okaloacoochee, Okaloacoochee Slough State Forest.

Our study area encompassed a large portion of the range of the breeding population of panthers in South Florida (Figure 1) south of the Caloosahatchee River and Lake Okeechobee that included habitats such as hardwood hammocks, cypress forests, pine flatwoods, freshwater marshes, prairies, and grasslands [19-21]. Anthropogenic land use included citrus, croplands, pastureland, rock mining, and areas of low- and high-density residential development [19,20]. Our study area can be categorized based on habitat types that vary longitudinally in the ratio of marsh/swamps to upland and wetland forests moving from the southern to the northern portion of the breeding range of panther. A small subpopulation of panthers persists in Everglades National Park [Everglades] in Southern Florida that is partially isolated from the core population by the semi-permeable barrier of the Shark River Slough (Figure 1). The core panther population is northwest of Everglades and is comprised of portions of Big Cypress National Preserve [Big Cypress] and Additional Land units of Big Cypress [Big Cypress Addlands], Picayune Strand State Forest/Fakahatchee Strand Preserve State Park [Picayune], and Florida Panther National Wildlife Refuge [Panther NWR]. The northern extent of the panther breeding range is comprised of a mix of public and private lands that includes Corkscrew Regional Ecosystem Watershed [Corkscrew] and Okaloacoochee Slough State Forest [Okaloacoochee]. The Caloosahatchee River is the northern border of the present breeding range of the Florida panther (Figure 1).

### Data collection

We used trained hounds to track and tree adult and subadult panthers for subsequent capture and radiocollaring by project personnel during concurrent research projects associated with management and conservation of panther from 2005 to 2013 [19,20]. We deployed five models of GPS collars produced by four manufacturers, including Advanced Telemetry Systems G2110 (Isanti, Minnesota, USA), Lotek GPS3300s (New Market, Ontario, Canada), Followit Tellus and Tellus-GSM (Lindesberg, Sweden), and Telonics TGW-3401 (Mesa, Arizona, USA). All GPS collars also were equipped with VHF beacons to allow relocations of specific panthers via aerial or ground telemetry. The GPS collection schedules varied (e.g., hourly, every 4 hours, every 7 hours) but were programmed into GPS collars to attempt to collect locations throughout the diel period.

To collect concurrent VHF locations of GPS-collared panthers, we used a Cessna 172 (Cessna Aircraft Company, Wichita, KS) equipped with a pair of directional antennas that were attached to a radio receiver via coaxial cable to estimate location of a VHF beacon in the GPS collar by selectively listening to radio signals from either or both antennas mounted to the struts of the wings and homing in on signal strength. We demarcated these locations using an application on a laptop computer synchronized with a GPS and loaded with satellite imagery to obtain Universal Transverse Mercator coordinates in flight. We conducted most telemetry flights between 0700 hours and 1100 hours 3 times per week (Monday, Wednesday, and Friday). We estimated the location of collars at fixed locations unknown to the observer (dropped collars, n = 2; mortalities, n = 23; and denning panthers, n = 20) during flights and determined VHF aerial telemetry location error to be 124 m [20]. The mean horizontal spatial accuracy for the GPS radiocollars used to collect data for this study was 34 m [19].

We collared 31 independent-aged panthers (12 females, 19 males) between February 2005 and February 2013 (Additional file 1). We monitored 25 of the 31 GPS-collared panthers concurrently with VHF technology for comparison of home range estimates using GPS versus VHF technology (Additional file 1). Age at capture ranged from 1.5 to 13.3 years and mean time collared was 278 days (49–610 days). We collected 75,758 locations over 101,865 attempts for an overall mean fix success rate of 74% (Additional file 1). Mean number of locations used to estimate annual home range was 1192 ± 1540 (SD) and 105 ± 29 (SD) for GPS and VHF technology, respectively.

### First-generation estimators

#### Local convex hull

We estimated utilization distributions with LOCO that produces bounded home ranges using a generalization of the minimum convex polygon method. Minimum convex polygon construction is applied to a subset of localized data in space using either *k* (k − 1 nearest neighbor), *r* (sphere of influence), or *a* (adaptive sphere of influence) of nearest neighbors [8,9]. The hulls are then sorted by size, ordered, and progressively unioned to construct a utilization distribution with hard boundaries (e.g., rivers, lakes) excluded. These hard boundaries often do not contain locations of animals so home range estimators should not extend beyond these hard boundaries as is often the case when using utilization distributions for parametric kernel methods [9]. For consistency across estimators, we only used *k* and the square root of the number of relocations for each individual for the value of k (http://locoh.cnr.berkeley.edu/rtutorial).

#### Single-linkage cluster

We estimated utilization distributions with the single-linkage cluster (SLCA) method that links 3 locations in clusters that minimizes the mean joining distance [22]. The clustering process is then a step process that finds the next nearest cluster based on minimum mean of the nearest-neighbor joining distance and the closest location, then the process stops when all relocations are assigned to the same cluster and fuse into a single home range [22].

#### Characteristic hull

We estimated utilization distributions with computation of the Delaunay triangulation to create characteristic hull (CHAR) polygons using a set of relocations then ordering triangles from smallest to largest [23]. The CHAR is similar in principal to LOCO and SLCA in that the number of potential characteristic hulls that can be generated from a set of points can extend to a minimum convex polygon estimate if no triangles are removed from Delaunay triangulation [23]. Unlike minimum convex polygon, CHAR produces estimates of home ranges with concave edges and encompasses fewer regions of space not used the by the animal when compared to minimum convex polygons.

#### Fixed kernel home range

We estimated utilization distributions using the fixed-KDE method because fixed kernel was considered most accurate compared with adaptive kernel [17,24]. We selected a location-based estimator using KDE with smoothing determined by the reference bandwidth (LKDE). We were unable to use biased cross-validation or least-squares cross-validation bandwidths for KDE because of the large number of duplicate locations and the propensity for numerous clusters of points [3].

### Second-generation estimator

We also estimated KDE using the bivariate plug-in bandwidth (PKDE) that performs well even when analyzing dependent data that is especially common from animals with locations collected with GPS technology [25]. First- and second-generation estimators do not include a temporal, error, or variance component in the estimation of home range.

### Third generation estimators

#### Movement-based kernel density estimator

We estimated utilization distributions with biased random bridges using the movement-based kernel density estimator (MKDE) that can incorporate time, distance, and habitat into estimates of home range [14,15]. Unlike traditional KDE, MKDE can integrate habitat-specific coefficients for movement, boundary constraints, and states of activity, thus improving estimates of home range [14,15]. We did not incorporate habitat into estimates of home range using MKDE for consistency because none of the other estimators we used incorporates this functionality. Furthermore, we set all parameters the same for each group of panthers as these values were based on GPS technology collection schedules thus complicating comparisons between studies or species with this method [14]. Due to constraints of MKDE for serially correlated data, we were not able to perform MKDE on datasets collected with VHF technology that resulted in <200 relocations for a given year with relocations separated by several days.

#### Brownian bridge movement model

We estimated utilization distributions using the BBMM that requires (1) sequential location data, (2) estimated error associated with location data, and (3) grid-cell size assigned for the output utilization distribution [12]. The BBMM is based on two assumptions: (1) location errors correspond to a bivariate normal distribution and (2) movement between successive locations is random [12]. The assumption of conditional random movement between paired locations becomes less realistic as the time interval increases [12].

#### Dynamic Brownian bridge movement model

We estimated utilization distributions using the dBBMM that requires the same parameters as BBMM [13]. The variance of the Brownian motion quantifies how diffusive or irregular the path of the animal is and is based on an average of all location data for BBMM. However, for dBBMM, the behaviorally distinct movement patterns are incorporated into estimates of home range and variance is determined using a moving window across each movement path and not simply averaging across the sample space of the animal as with BBMM [13].

### Home range estimate criteria

We estimated annual home range for each panther that had >50 locations for each year for both GPS and VHF datasets with year defined as a calendar year from 1 January to 31 December. All estimators were calculated in R using the packages *adehabitatHR* (LKDE, MKDE, LOCO, SCLA, CHAR; [26]), *ks* (PKDE; [27]), *BBMM* (BBMM; [28]), and *move* (dBBMM; [13]). We modified R code provided in Cumming and Cornelis [10] to: estimate AUC, estimate LOCO directly in R, and included 4 additional estimators (CHAR, PKDE, BBMM, dBBMM) not evaluated previously. Due the changing parameters for each estimator, location data was imported, manipulated, and adapted to the appropriate package for each estimator in a loop function in R (Additional file 2).

### Area-under-the-curve

Due to scale dependency for AUC-based assessment, all home ranges were estimated on reference grids that were 100 × 100 m at identical grain and extent around each animal [10]. Comparisons of AUC for estimators of home range across species that occupied varying degrees of spatial extents and movements across the landscape would be difficult and would require additional considerations so only one species was considered in our analysis [10]. We calculated AUC in R using the *caTools* package where AUC ranges between 0.5 and 1.0 with 1.0 indicating relocations fit more accurately to the resulting isopleths of the estimated home range [10]. All values of AUC were computed for each individual for each of the 8 estimators of home range using a script in R that produces figures of home range contours and outputs AUC and associated data (Additional file 2).

### Statistical analysis

We performed a Kruskal-Wallis nonparametric analysis-of-variance to determine if differences occurred between GPS and VHF technology among AUC for all estimators. We then used pairwise t-tests with a Bonferroni correction on AUC because a difference occurred between technology and estimator combinations (Kruskal-Wallis chi-squared = 573.99, P < 0.001).

We fit linear mixed models with animal identification as a random effect to the logit-transformed response variable (AUC) of home ranges estimated by GPS technology. Fixed effects were covariates that have been suggested to influence the accuracy of estimating home range that included 5 covariates: estimator type, fix success, study area, GPS collection schedule, and number of locations used to estimate home range [29,30]. We set the reference level of the estimator type to SLCA because it was considered the least preferred estimator due to length of time to provide estimate and >1,000 locations often failed to produce home ranges. Number of locations was placed into 4 categories (1) <100, (2) 101–500, (3) 501–1000, and (4) >1000 that were within the ranges of sample sizes for estimating annual/seasonal home ranges used in previous research [4,9,10]. Fix success was determined from the number of locations successfully acquired by the GPS divided by the number of locations attempted. Since the collection schedules for GPS collars varied, we delineated 3 categories that included location collections every (1) hour or less (*hourly*), (2) 2–4 hours (*four*), and (3) 7–14 hours (*seven*). Study area was categorical and used as a proxy for habitat interference in acquiring a GPS location or influencing accuracy and represented a continuum of generally more open marsh landscape in South Florida to more upland and wetland forested habitats in the northern portion of the breeding range (Figure 1; [19,20]). We identified 12 models *a priori* with various combinations of the 5 covariates that may influence size of home range estimates as determined by AUC (Table 1). We performed model selection using the second-order variant of Akaike’s Information Criteria (AICc), which accounts for overdispersion and small sample size, to select the most parsimonious model [31]. We did not include any interaction terms to prevent over-parameterization of the model [31]. Models were considered a candidate if they had a ΔAIC_c_ <4.0 and we assessed the degree that 95% confidence intervals of parameter estimates overlapped zero to support AIC as evidence of important effects [31].

**Table 1:** **Model selection results for the candidate set of models investigating the effect of covariates on area-under-the-curve for 8 estimators of home range for Florida Panther from 2005 to 2013 in Southern Florida, USA**

**Model terms**	**K**	**AIC**_**c**_	**∆AIC**_**c**_	**Weights**
Estimator + (1| animal)	10	786.06	0.00	1.0
Estimator + Percent fix success + GPS schedule + Number of locations + Study Area + (1| animal)	22	807.98	21.92	0.00
Intercept	3	1098.29	312.23	0.00
GPS schedule + (1| animal)	5	1103.15	317.09	0.00
Percent fix success + (1| animal)	5	1104.77	318.70	0.00
Number of locations + (1| animal)	6	1105.93	319.86	0.00
Percent fix success + GPS schedule + (1| animal)	7	1107.46	321.39	0.00
GPS schedule + Number of locations + (1| animal)	8	1111.06	324.99	0.00
Study Area + (1| animal)	8	1111.12	325.06	0.00
Percent fix success + GPS schedule + Number of locations + (1| animal)	10	1115.40	329.33	0.00
Number of locations + Study Area + (1| animal)	11	1118.28	332.21	0.00
Number of locations + GPS schedule + Study Area + (1| animal)	13	1122.14	336.07	0.00

The term in parenthesis represents the random effect in our model.

## Results

**Figure 2:**
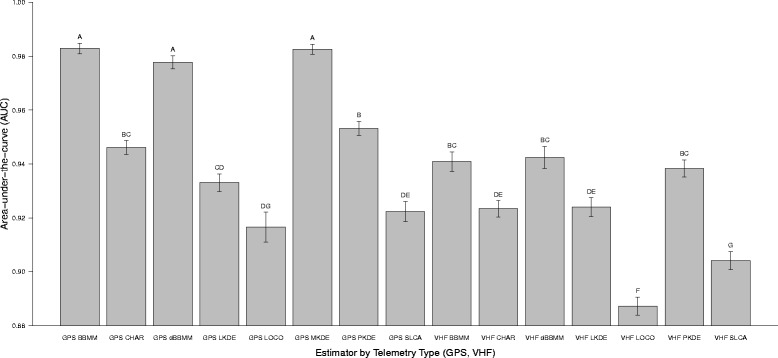
Mean (± SE) area-under-the-curve for estimators of home range collected with global positioning system (GPS) technology and very high frequency (VHF) technology. Different numbers above bars indicated differences between estimators at *P* = 0.05.

Mean AUC differed among several estimators and technology type (Kruskal-Wallis *x*^*2*^ = 573.99, *df* =14, *P* < 0.001) with the highest AUC consistently occurring for GPS compared to VHF technology (Figure 2). Mean AUC for GPS technology was highest for BBMM (mean = 0.982 ± 0.01 (SD)) and lowest for LOCO (mean = 0.916 ± 0.03 (SD); Figure 2). Mean AUC for VHF technology was highest for dBBMM (mean = 0.942 ± 0.03 (SD)) and lowest for LOCO (mean = 0.887 ± 0.02 (SD); Figure 2) but we were not able to estimate MKDE for VHF technology due to the irregular temporal duration and distances between locations with this method.

**Figure 3:**
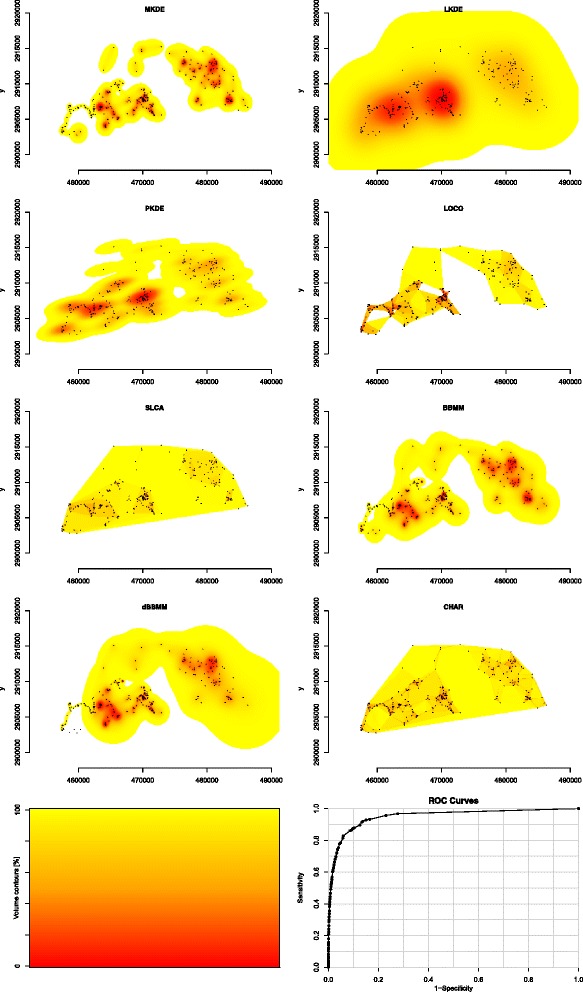
Example of area-under-the-curve showing differences in isopleths for 8 estimators of home range for Florida panther 185 collected with global positioning system (GPS) technology in 2011. Movement-based kernel density estimator (MKDE), location-based kernel density estimator using h_plug-in_ smoothing (PKDE), location-based kernel density estimator using h_ref_ smoothing (LKDE), Brownian Bridge Movement Model (BBMM), polygon-derived single-linkage cluster analysis (SLCA), polygon-derived characteristic hull (CHAR), polygon-derived local convex hull (LOCO), and dynamic Brownian Bridge Movement Model (dBBMM). Volume contours reflects isopleths from 0 to 100% (bottom left) and an example ROC curve (bottom right).

Our model with the most support included only the 8 estimator types with no additional covariates included (*w*_*i*_ = 1.0; Table 1). The global model that included all 5 covariates was the next most supported model but had a ΔAIC_c_ >4.0 so was not considered further (Table 1). Parameter estimates identified an increase in AUC for the BBMM, dBBMM, and MKDE estimators but a decrease with LOCO (Table 2).

**Table 2:** **Parameter estimates, standard error (SE), and 95% confidence intervals (CI) for parameters in the most supported model investigating the effect of covariates on the area-under-the-curve for Florida panther equipped with GPS technology from 2005 to 2013 in Southern Florida, USA**

**Parameter**	**Estimates**	**SE**	**CI**
Intercept	2.550	0.108	2.337 to 2.762
BBMM	1.856	0.132	1.598 to 2.134
CHAR	0.368	0.147	0.079 to 0.657
dBBMM	1.474	0.132	1.216 to 1.732
LKDE	0.152	0.132	−0.106 to 0.410
LOCO	−0.083	0.141	−0.387 to 0.165
MKDE	1.8642	0.132	1.606 to 2.122
PKDE	0.5673	0.132	0.309 to 0.825

## Discussion

Data collected with VHF technology (i.e., intervals between successive locations spanning days to weeks) to estimate home range using third-generation estimators should be critically reviewed based on our comparison using concurrent GPS location data (i.e., typically <12 hours between locations) from the same animals. The AUC differed for estimators of home range determined using data collected with GPS compared to estimates of home range derived from concurrent VHF locations, which were typically estimated with 10% fewer locations (Additional file 1). Although GPS technology is more expensive to purchase initially, the high costs of aerial/ground-based location acquisition and the gains in data reliability, quantity, and reduced error far outweigh the disadvantages of relying on <100 locations collected with VHF technology, which then assumes that comparatively few locations represents the home range or space use of an animal [32].

There were clear differences in the fit of location data to isopleths of each home range estimator as determined through AUC using GPS technology. Kernel-based or Brownian bridge-based estimators appear to have the best fit to the data over polygon-derived estimators such as local convex hull and single-linkage cluster. Furthermore, polygon-derived estimators were limited in their capabilities of handling large GPS datasets over 1,000 locations and large voids in space use within the extent of a home range prevented estimation of home range for some panthers when using these estimators. Local-convex hull was considered an improved method that could identify hard boundaries such as roads or bodies of water and would exclude large unused space within the home range [8,9] but our results support previous studies that suggested local-convex hull has considerable limitations for sizeable datasets collected with GPS technology [10,33,34].

Estimators that incorporate a temporal component appeared to be the most reliable regardless of whether kernel-based or Brownian bridge-based algorithms were used. Researchers have identified numerous components of GPS data collection that should improve estimation of home range such as consistency in duration between locations (i.e., collection schedule), GPS error, and movement-specific parameters that could vary by individual [12-14]. Location-based kernel density estimators that are not able to incorporate temporal duration (i.e., LKDE, PKDE) were comparable to polygon-derived estimators with lower mean and greater variability in AUC further strengthening the suggestions that incorporation of a temporal component within an estimate of home range may improve resulting isopleths. Although the second-generation estimator (PKDE) yielded higher AUC than the first-generation estimator (LKDE), variability in AUC indicated that second-generation estimators of KDE may be less appropriate now that time and space can be incorporated into estimation of home range with GPS technology. These third-generation estimators of home range (e.g., MKDE, dBBMM) extend beyond traditional KDE by incorporating time, space, and animal-specific parameters in addition to habitat-specific movement vectors such as in MKDE. Additional estimators, such as time-geographic density estimation and time-local convex hull, may also prove to be more robust at providing reliable estimates of home range [35,36], although they have yet to be implemented in R or were not evaluated.

**Figure 4:**
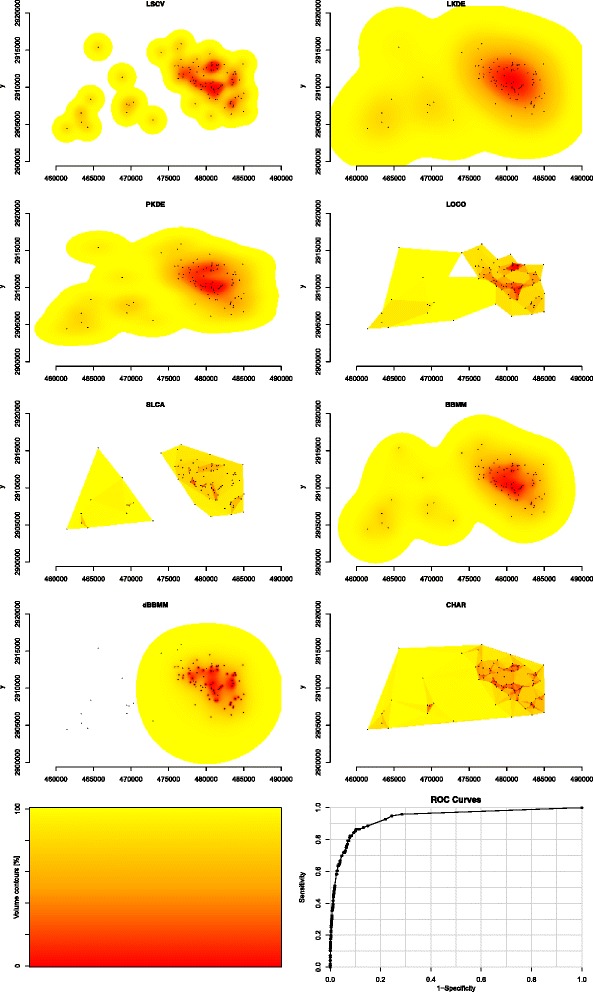
Example of area-under-the-curve showing differences in isopleths for 8 estimators of home range for Florida panther 185 collected with concurrent Very High Frequency (VHF) technology in 2011. Movement-based kernel density estimator (MKDE) was not able to be estimated with VHF technology so was replaced with location-based kernel density estimator using least squares cross-validation smoothing (LSCV). Location-based kernel density estimator using h_plug-in_ smoothing (PKDE), location-based kernel density estimator using h_ref_ smoothing (LKDE), Brownian Bridge Movement Model (BBMM), polygon-derived single-linkage cluster analysis (SLCA), polygon-derived characteristic hull (CHAR), polygon-derived local convex hull (LOCO), and dynamic Brownian Bridge Movement Model (dBBM). Volume contours reflects color scheme for isopleths from 0 to 100% (bottom left) and an example ROC curve (bottom right).

A caveat in our study is that we evaluated annual home ranges but we chose not to evaluate the influence of the extent of location data across the landscape and resulting estimation of home range on AUC. The variability in our data for some estimators may be attributed to using annual home range that incorporate animal-specific movements (e.g., seasonal migration, long-distance exploratory movements), when they are actually present, as opposed to shorter-duration seasonal home ranges. This issue may have resulted in poorer estimates for location-based KDE or polygon-derived estimators than third-generation estimators due to over-estimation of home ranges as previously reported (Figures 3 and 4; [16,29]). Studies using location-based kernel estimators traditionally separated locations by season or pre-defined periods to avoid over-estimation of size of home range, however, third-generation-based estimators are able to account for large movements across the landscape to more accurately reflect a home range that may span several seasons or geographical extents. Furthermore, estimators of home range that incorporate animal-specific data or duration between locations into estimates of home range intuitively would appear to fit a movement trajectory better than location-based or polygon-derived estimators that “fill in” the gaps between clusters of locations [3,14].

## Conclusions

Our results indicated that locations collected with GPS technology consistently performed better than those collected with VHF technology to estimate home range and use of the latter in home range studies should be avoided. All estimators of home range performed better using GPS-based locations likely because several variables can affect estimation of home range using GPS location data (e.g., sample size of locations, duration between locations) and these variables can be directly accounted for within third-generation estimators. Development of these third-generation estimators were a result of GPS datasets and previous research suggesting that location-specific parameters and landscape characteristics influenced accuracy of estimates of size of home range [3,34,37]. Our focal species exhibited relatively large home ranges, but these ranges can have extensive areas that may not be used such as urban development or fenced roadways. Estimators that more accurately reflect the utilization of the landscapes by species, especially those that are endangered, is important to developing conservation initiatives that will assist with recovery. The extent of the available landscape a species travels across during a season or year should be considered and likely influenced AUC in our study. Further examination by study area across the range of a species or multiple species should be explored to further assess landscape-level covariates that may influence selection and accuracy of third-generation estimators of home range. The availability of third-generation estimators and inconsistency of first- and second-generation estimators in determining size of home range along a range of sample sizes and individual panther in our study would appear to justify exclusive use of and evaluation of third-generation methods as estimators of home range using GPS technology.

## Additional files

Additional file 1: Table S1. Sample size of locations for Florida panthers fitted with global positioning system technology (very high frequency technology), study area, collection schedule, and percent fix success from February 2005 to February 2013 in South Florida, USA. An asterisk indicates the panthers that were tracked concurrently using VHF technology from an aircraft. Percent fix success (Success) represents the total number of locations collected divided by the number of attempts to obtain locations from satellites. Estimator abbreviation refer to: location-based kernel density estimator using reference bandwith smoothing (LKDE), location-based kernel density estimator using plug-in smoothing (PKDE), movement-based kernel density estimator (MKDE), polygon-derived single-linkage cluster analysis (SLCA), polygon-derived local convex hull (LOCO), polygon-derived characteristic hull (CHAR), Brownian Bridge Movement Model (BBMM), and dynamic Brownian Bridge Movement Model (dBBM). Additional file 2: Table S2. Script for Program R to determine home range for each of 8 estimators with subsequent analysis of fit using area-under-the-curve.

## Abbreviations

GPS: Global positioning system
VHF: Very high frequency
KDE: Kernel density estimator
LOCO: The local convex hull nonparametric kernel method
SLCA: Single-linkage cluster
CHAR: Characteristic hull
LKDE: Kernel density estimator with reference bandwidth
PKDE: Kernel density estimator with plug-in bandwidth
MKDE: Movement-based kernel density estimator
BBMM: Brownian bridge movement models
dBBMM: Dynamic Brownian bridge movement models
AUC: Area-under-the-curve
