# Supplementary material for: Is there a single best estimator? Selection of home range estimators using area-under-the-curve
Source: Mov Ecol. 2015 Apr 16;3(1):10. doi: 10.1186/s40462-015-0039-4 (PMC4429481; doi:10.1186/s40462-015-0039-4)
Supplement: Additional file 1: Table S1. — Sample size of locations for Florida panthers fitted with global positioning system technology (very high frequency technology), study area, collection schedule, and percent fix success from February 2005 to February 2013 in South Florida, USA. An asterisk indicates the panthers that were tracked concurrently using VHF technology from an aircraft. Percent fix success (Success) represents the total number of locations collected divided by the number of attempts to obtain locations from satellites. Estimator abbreviation refer to: location-based kernel density estimator using reference bandwith smoothing (LKDE), location-based kernel density estimator using plug-in smoothing (PKDE), movement-based kernel density estimator (MKDE), polygon-derived single-linkage cluster analysis (SLCA), polygon-derived local convex hull (LOCO), polygon-derived characteristic hull (CHAR), Brownian Bridge Movement Model (BBMM), and dynamic Brownian Bridge Movement Model (dBBM). [file 40462_2015_39_MOESM1_ESM.pdf]

		Study			Estimator							
ID	Sex	Area[a]	Schedule	Success	LKDE	PKDE	MKDE	SLCA	LOCO	CHAR	BBMM	dBBM
FP048	F	FPNWR	Hourly	74.4%	9346 (174)	9346 (176)	9346 (NA)	NA[b] (NA)	9944 (176)	NA (176)	9344 (174)	9344 (176)
FP094*	F	EVER	Hourly	80.9%	7436 (309)	7436 (309)	7436 (NA)	1209 (309)	8230 (155)	1209 (155)	7433 (154)	7435 (155)
FP110*	F	OKS	Seven	66.6%	1154 (836)	1154 (836)	1154 (NA)	1155 (836)	1155 (716)	1155 (716)	1151 (710)	1154 (716)
FP113*	F	FPNWR	Seven	69.7%	743 (1050)	743 (1050)	743 (NA)	743 (1050)	743 (1254)	125 (1050)	741 (1042)	742 (1050)
FP121*	F	BCNP-AL	Seven	58.7%	366 (69)	366 (69)	366 (NA)	366 (69)	366 (69)	366 (69)	365 (68)	365 (69)
FP128*	F	BCNP-AL	Seven	55.4%	438 (249)	438 (249)	438 (NA)	438 (249)	438 (249)	438 (249)	437 (246)	437 (249)
FP130*[c]	M	OKS[b]	Hourly	81.0%	7241 (175)	7241 (175)	7241 (NA)	NA (175)	8249 (175)	NA (175)	7239 (173)	7238 (175)
FP131*	M	FPNWR	Hourly	77.8%	6307 (335)	6307 (335)	6307 (NA)	798 (445)	7356 (NA)	798 (NA)	6305 (NA)	6306 (NA)
FP135*	M	FPNWR	Seven	60.9%	527 (211)	527 (211)	527 (NA)	528 (211)	528 (NA)	528 (NA)	526 (NA)	527 (NA)
FP137*[c]	M	OKS	Four	72.1%	2038 (307)	2038 (307)	2038 (NA)	2038 (307)	2038 (307)	204 (181)	2036 (304)	2038 (307)
FP137 [d]	M	OKS	Hourly	89.1%	1949 (ND[e])	1949 (ND)	1945 (ND)	1949 (ND)	1949 (ND)	433 (ND)	1947 (ND)	1947 (ND)
FP139	M	OKS	Hourly	68.4%	2332 (ND) [d]	2332 (ND)	2331 (ND)	ND (ND)	ND (ND)	ND (ND)	2331 (ND)	2331 (ND)
FP142*	F	EVER	Four	69.9%	1529 (234)	1529 (234)	1529 (NA)	1529 (234)	1529 (234)	111 (234)	1527 (232)	1528 (234)
FP143*	M	BCNP-AL	Four	58.9	690 (195)	690 (195)	690 (NA)	690 (195)	690 (195)	690 (195)	689 (193)	689 (195)
FP146*	M	PSSF	Seven	67.0	1914 (306)	1914 (306)	1914 (NA)	1913 (306)	1913 (306)	819 (306)	1911 (303)	1912 (306)
FP148*	F	PSSF	Seven	54.6	582 (562)	582 (562)	582 (NA)	583 (562)	583 (562)	63 (562)	580 (557)	582 (562)
FP149*	F	PSSF	Seven	72.2	283 (56)	283 (56)	283 (NA)	283 (56)	NA (56)	283 (56)	282 (55)	283 (56)
FP155	M	OKS	Hourly	73.5	4672 (ND)	4672 (ND)	4672 (ND)	618 (ND)	5687 (ND)	618 (ND)	4670 (ND)	4671 (ND)

		Study			Estimator							
ID	Sex	Area[a]	Schedule	Success	LKDE	PKDE	MKDE	SLCA	LOCO	CHAR	BBMM	dBBM
FP156*	M	PSSF	Four	29.4	468 (263)	468 (263)	468 (NA)	468 (263)	NA (263)	468 (263)	466 (260)	467 (263)
FP157	M	CREW	Hourly	67.4	1715 (ND)	1715 (ND)	1715 (ND)	1715 (ND)	ND (ND)	194 (ND)	1713 (ND)	1714 (ND)
FP159*	M	CREW	Hourly	84.5	381 (164)	381 (164)	376 (NA)	381 (164)	381 (NA)	381 (NA)	380 (162)	376 (NA)
FP160*	F	OKS	Four	43.7	481 (101)	481 (101)	481 (NA)	481 (101)	NA (101)	481 (101)	480 (100)	480 (101)
FP165*	M	OKS	Hourly	77.1	702 (57)	702 (57)	642 (NA)	702 (57)	702 (57)	702 (57)	700 (56)	678 (57)
FP167*	M	BCNP-AL	Hourly	80.1	4702 (91)	4702 (91)	4702 (NA)	NA (91)	NA (91)	NA (91)	4701 (90)	4701 (91)
FP173*	M	CREW	Four	43.1	694 (112)	694 (112)	629 (NA)	694 (112)	NA (112)	694 (112)	693 (111)	629 (112)
FP174	M	PSSF	Hourly	88.3	322 (ND)	322 (ND)	321 (ND)	322 (ND)	322 (ND)	322 (ND)	321 (ND)	321 (ND)
FP183*	M	PSSF	Hourly	91.5	2780 (303)	2780 (303)	2721 (NA)	2780 (303)	1801 (NA)	979 (NA)	2778 (300)	2737 (NA)
FP185*	F	FPNWR	Hourly	87.1	1434 (100)	1434 (100)	1388 (NA)	1434 (100)	429 (100)	1434 (100)	1432 (99)	1415 (100)
FP188*	F	CREW	Four	59.1	606 (78)	606 (78)	582 (NA)	606 (78)	606 (78)	606 (78)	605 (77)	582 (78)
FP189*	M	FPNWR	Hourly	86.3	4734 (91)	4734 (91)	4689 (NA)	NA (91)	NA (91)	NA (91)	4733 (90)	4717 (91)
FP193*	M	PSSF	Four	59.0	1356 (311)	1356 (311)	1317 (NA)	1356 (311)	1356 (NA)	1356 (NA)	1354 (308)	1317 (NA)

[a] EVER, Everglades National Park; BCNP-AL, the Additional Land units of Big Cypress National Preserve; PSSF, Picayune Strand State Forest/Fakahatchee Strand Preserve State Park; FPNWR, Florida Panther National Wildlife Refuge; CREW, Corkscrew Regional Ecosystem Watershed; and OKS, Okaloacoochee Slough State Forest.
[b] NA refers to Not Available because the number of locations or distribution of locations prevented estimation of home range.
[c] FP130 was initially fitted with a VHF collar when captured at OKS. He was subsequently recaptured north of OKS and fitted with a GPS collar. We still categorized his study area as OKS given that he used habitat at the northern extreme of the breeding range and beyond (see Fig. 1)
[d] Due to collar failure after one year of data collection at 2 hour intervals, FP137 was collared with a new collar that collected locations each hour and was considered a separate animal for purposes of our study.
[e] ND refers to No Data because some panthers were not tracked concurrently with very high frequency technology.
